# Assessing a novel immuno-oncology-based combination therapy: Ipilimumab plus electrochemotherapy

**DOI:** 10.1080/2162402X.2015.1008842

**Published:** 2015-05-22

**Authors:** Nicola Mozzillo, Ester Simeone, Lucia Benedetto, Marcello Curvietto, Diana Giannarelli, Giusy Gentilcore, Rosa Camerlingo, Mariaelena Capone, Gabriele Madonna, Lucia Festino, Corrado Caracò, Gianluca Di Monta, Ugo Marone, Massimiliano Di Marzo, Antonio M Grimaldi, Stefano Mori, Gennaro Ciliberto, Paolo A Ascierto

**Affiliations:** 1 Melanoma and Sarcoma Surgery Unit; Istituto Nazionale Tumori Fondazione “G. Pascale”; Naples, Italy; 2 Melanoma, Cancer Immunotherapy, and Innovative Therapy Unit; Istituto Nazionale Tumori Fondazione “G. Pascale”; Naples, Italy; 3 Statistical Unit; Regina Elena National Cancer Institute; Rome, Italy; 4 Scientific Direction; Istituto Nazionale Tumori Fondazione “G. Pascale”; Naples, Italy

**Keywords:** biomarker, Electrochemotherapy, immuno-oncology, ipilimumab, melanoma

## Abstract

Melanoma is responsible for most skin cancer-related deaths and is one of the most common cancers diagnosed in young adults. In melanoma, tumors can become established by activation of the negative regulator of cytotoxic T lymphocytes (CTLs), CTL antigen-4 (CTLA-4). Ipilimumab blocks the interaction of CTLA-4 with CD80/CD86 and augments T-cell activation and proliferation. In electrochemotherapy (ECT), local application of short high-voltage pulses renders cell membranes transiently permeable to chemotherapeutic drugs. The combination of ipilimumab and ECT may be beneficial for the treatment of metastatic melanoma; however, no prospective data are available to date. Here, we report the retrospective analysis of patients treated with ipilimumab in an expanded access program (EAP) who also received ECT. Fifteen patients with previously treated metastatic melanoma who received ipilimumab 3 mg/kg every three weeks for four cycles and underwent ECT for local disease control and/or palliation of cutaneous lesions with bleomycin 15 mg/m^2^ after the first ipilimumab infusion were included in the analysis. Over the study period, a local objective response was observed in 67% of patients (27% complete response [CR] and 40% partial response [PR]). According to immune-related response criteria, a systemic response was observed in nine patients (five PR and four stable disease [SD]), resulting in a disease control rate of 60%. Evaluation of circulating T-regulatory (T-reg) cells demonstrated significant differences between responders and non-responders. Overall, treatment was well-tolerated and without notable toxicity. In conclusion, the combination of ipilimumab and ECT appears to be beneficial to patients with advanced melanoma, warranting further investigation in prospective trials.

## Introduction

In 2012, the worldwide annual incidence of melanoma was approaching 0.25 million and resulted in >55,000 deaths (estimated figures from GLOBOCAN 2012).^1^ Despite increasing awareness through educational campaigns and other initiatives, these figures are predicted to continue to rise.^1^ This is particularly true for adults <30 years old, with melanoma recognized as one of the commonest cancers diagnosed in young adults (ages 15–29 years).^2^ Furthermore, the five-year relative survival rate for advanced-stage melanoma is a dismal 15%.^3^ These statistics highlight the need for continuing education, new agents and novel approaches to treatment for patients with melanoma.

Until recently, there has been a dearth of effective agents for metastatic melanoma. However, the last few years have seen the approval of novel agents, including immunotherapies. There are several reasons that suggest immuno-oncology has a place in this setting. Melanomas initiate an immune response, which triggers infiltrating CTLs to recognize and destroy the cancer cells. Unfortunately, neoplastic cells can evade the body's normal immunosurveillance systems and thereby avoid their subsequent removal, a process which can lead to a tumor becoming established. Evasion of the immune system can be achieved by various means including via activation of checkpoint receptors; for example, the receptors that bind CTLA-4 or programmed death ligand-1 (PD-L1) and which down regulate T-cell activity.^4,5^

Ipilimumab is a fully human monoclonal antibody (IgG1) that promotes T-cell-mediated antitumor activity in patients with melanoma by blocking the interaction of CTLA-4 with CD80/CD86 and augmenting T-cell activation and proliferation.^6,7^ Ipilimumab was the first agent approved for the treatment of metastatic melanoma that has been shown to achieve a significant overall survival benefit.^8,9^

Despite the breakthrough made with ipilimumab as monotherapy, the current challenge is to assess whether outcomes can be further improved by combining with other treatment modalities. Ipilimumab is currently being assessed in combination with chemotherapy, targeted agents and other immunotherapy agents with different mechanisms of action. For example, a recent trial combining ipilimumab with nivolumab (a monoclonal antibody against PD-1 receptor) suggested an improved response versus either as monotherapy,^10,11^ pending of confirmatory results from the CA209-067-randomized clinical trial (NCT01844505).

Ipilimumab is also being assessed in combination with various local therapies. For example, there is also evidence that ipilimumab combined with radiotherapy (RT) may have therapeutic benefit.^12-14^ Similarly, it has been reported that ipilimumab in combination with the HSV-1-derived oncolytic immunotherapy, talimogene laherparepvec (T-VEC), may result in improved response rates compared with either agent alone.^15^ Another option may be to combine immunotherapy with ECT. ECT is a tumor ablation modality which consists of the local application of short duration (˜5 KHz for 100 μs) but high-voltage (several hundred Vcm^−1^) pulses which transiently increase cell membrane permeability to cytotoxic chemotherapeutic drugs, such as bleomycin or cisplatin.^16,17^ A particular benefit of ECT is that it facilitates the treatment of tumor nodules occurring in the proximity of important and often vulnerable structures such as blood vessels and nerves where surgery is not possible.

Clinical evidence supporting the use of ECT for local control of metastatic melanoma with superficial lesions is now emerging. Studies investigating the effectiveness of ECT in patients with advanced melanoma have recently been reported and suggest overall response rates ranging from 55 to 99%.^18^ A meta-analysis of 44 studies involving 1894 tumors demonstrated that addition of ECT significantly increased efficacy compared with cytotoxic systemic therapy alone (*P* < 0.001).^19^ Although intratumoural administration of bleomycin was significantly more effective than intravenous administration, intravenous use combined with ECT was more effective than bleomycin alone. In addition, other studies have also reported long-lasting tumor responses and notable tumor control rates in patients with melanoma who received ECT.^20, 21^

The combination of two distinct treatment modalities, such as ECT and immunotherapy could be an intriguing approach for the treatment of patients with metastatic melanoma. In fact, early preclinical data provide evidence for involvement of the immune system in the response to this modality. For example, complete remission was observed in studies in immune-competent mice but not in immune-deficient mice.^22-25^ It is possible, therefore, that tumor-associated antigens may be recognized by inflammatory cells that migrate to the nodule upon treatment. This may be as a result of ECT-related cellular damage that release tumor antigens which the immune system can react to.

**Figure 1.**
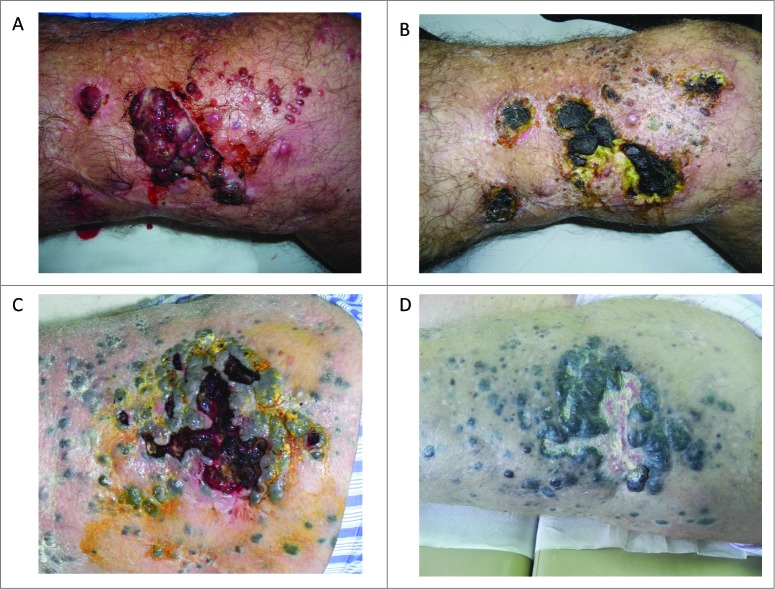
Bleeding cutaneous metastases of melanoma which were treated with palliative electrochemotherapy. (**A**) (**C**) cutaneous lesions before ECT treatment; (**B**) (**D**) the same lesions after ECT treatment. ECT = Electrochemotherapy.

Understanding the potential benefits of combining ipilimumab and ECT could be important in developing optimal treatment strategies for patients with advanced melanoma. However, no prospective data using this combination are available to date. Here, we report a retrospective analysis of 15 patients treated with ipilimumab and ECT (plus systemic bleomycin), enrolled in the ipilimumab EAP at the National Cancer Institute “Fondazione G. Pascale” in Napoli, Italy. This retrospective analysis was not a formal, planned trial but was possible due to the occasional use of ECT for palliative loco-regional control of bleeding (**Fig. 1**) or painful lesions in patients included in the ipilimumab EAP. The analysis was conducted to retrospectively evaluate the benefit of treatment for patients in terms of immune-related disease control rate (irDCR). In the same analyses, evaluation of circulating T-reg cells as potential predictive biomarkers for response to this treatment combination was performed.^26^

## Results

### Patients and treatment duration

Between March 2011 and May 2013, 15 patients with advanced melanoma were treated with ipilimumab followed by ECT for loco-regional control. Median age was 61 years (range 40–79 years) and 10 (67%) patients were female. Seven patients had stage IIIc disease and eight had stage IV M1c disease. Baseline lactate dehydrogenase (LDH) was elevated in four patients (27%). Patient characteristics are reported in **Table 1**.

**Table 1.** Baseline characteristics of patients

Characteristic	*N* = 15
Median age, years (range)	61 (40–79)
Male/female, *n* (%)	5/10,50
Disease stage, *n* (%)	
IIIc	7 (47)
IV M1a	0 (0)
IV M1b	0 (0)
IV M1c	8 (53)
ECOG PS, *n* (%)	
0	15 (100)
1	0
Tumor subtype, *n* (%)	
Cutaneous	14 (93)
Mucosal	0 (0)
Ocular	1 (7)
Unknown	0 (0)
Baseline LDH, *n* (%)	
Elevated	4 (27)
Normal	11(73)
BRAF^V600^ mutation positive, *n*/*n* (%)	4/15 (27)
NRAS^Q61R^ mutation positive, *n*/*n* (%)	1/15 (7)
BRAF/NRAS WT, *n*/*n* (%)	10/15 (66)

ECOG PS, Eastern Cooperative Oncology Group performance status; LDH, lactate dehydrogenase.

All patients had received prior therapy for metastatic disease (**Table 2**), which included dacarbazine (*n* = 8), temozolomide (*n* = 5), vemurafenib (*n* = 1) and fotemustine (*n* = 1). Fourteen patients had cutaneous melanoma and one had ocular melanoma. Median time from diagnosis to documentation of metastatic disease was 24 months (range 6–96 months). Metastases in the skin and/or subcutaneous tissue were observed in all patients. Other disease sites included the liver (*n* = 4), lung (*n* = 2) and lymph nodes (*n* = 2).

**Table 2.** Disease characteristics and response

Patient	Date of melanoma diagnosis	Date metastatic disease was diagnosed	Location of metastatic disease	Prior therapy for metastatic disease	Local response to ECT	Systemic response
1	Aug-10	Jan-12	Liver, skin right thigh	Temozolamide	CR	PR
2	Jun-09	May-12	Liver, skin lower right leg	Dacarbazine	PR	PR
3	Jun-10	Jan-11	Skin/subcutaneous tissue on lower right leg	Temozolamide	PR	SD
4	Apr-07	Feb-11	Skin/subcutaneous tissue on lower right leg	Dacarbazine	PD	SD
5	May-09	Mar-11	Skin/subcutaneous tissue on lower left leg	Temozolamide	PR	PR
6	May-09	Jan-11	Lymph nodes, skin on lower right leg	Vemurafenib	PR	SD
7	Apr-04	Mar-12	Skin/subcutaneous tissue on lower left leg	Dacarbazine	PR	PD
8	Jul-08	Feb-12	Liver, skin on head/face	Fotemustine	PR	PD
9	Sep-10	Mar-11	Lung, skin on head/face	Dacarbazine	CR	PR
10	Apr-07	Sep-10	Skin/subcutaneous tissue on left chest	Temozolamide	CR	PR
11	Oct-10	Jul-11	Skin/subcutaneous tissue on lower right leg	Temozolamide	PD	PD
12	Apr-08	Apr-13	Lung, left cheek on mucosal tissue	Dacarbazine	CR	SD
13	Mar-08	Oct-11	Lymph nodes, lung, skin on right leg	Dacarbazine	PD	PD
14	Mar-11	Mar-13	Liver, skin on left leg	Dacarbazine	PD	PD
15	May–11	Jan-13	Skin/subcutaneous tissue on lower left leg	Dacarbazine	PD	PD

CR = complete response; PD = progressive disease; PR = partial response; SD = stable disease.

All patients received all four infusions of ipilimumab therapy and one treatment with ECT to target cutaneous and subcutaneous tumors. The total dose of bleomycin (15 mg/m^2^) received was either 20 mg (*n* = 4), 25 mg (*n* = 4), or 30 mg (*n* = 7).

### Efficacy

#### Local response to treatment

No local responses were observed at the time of the first assessment (Week 2). Over the assessment period, a local objective response was observed in 10/15 patients (objective response rate [ORR] of 67%: four patients with CR and six patients with PR). The remaining five patients (33%) had local progressive disease (PD). All six PRs were observed at Week 4 following the second dose of ipilimumab. Among the four CRs, one was observed at Week 4 and three at Week 7. All CRs were maintained at the end of the 12-week ipilimumab treatment period.

### Systemic response to treatment

Systemic response, according to immune-related response criteria (irRC), was observed in 9/15 patients, giving an irDCR of 60%. Of these nine patients, five (56%) had a PR and four (44%) had SD >3 months. At Week 12, six patients (40%) had PD. All nine patients with a systemic response were still alive at the time of this analysis, with a median follow up of 16.2 months (range 5–30 months).

All five patients who had a systemic response (non ECT-treated lesions) also had a local response (three with CR and two with PR). Of patients with systemic SD >3 months, one had a local CR and two had a local PR.

Overall survival (OS) rate was 93.3% at 6 months and 86.2% at 12 months.

### Biomarker analyses

**Figure 2.**
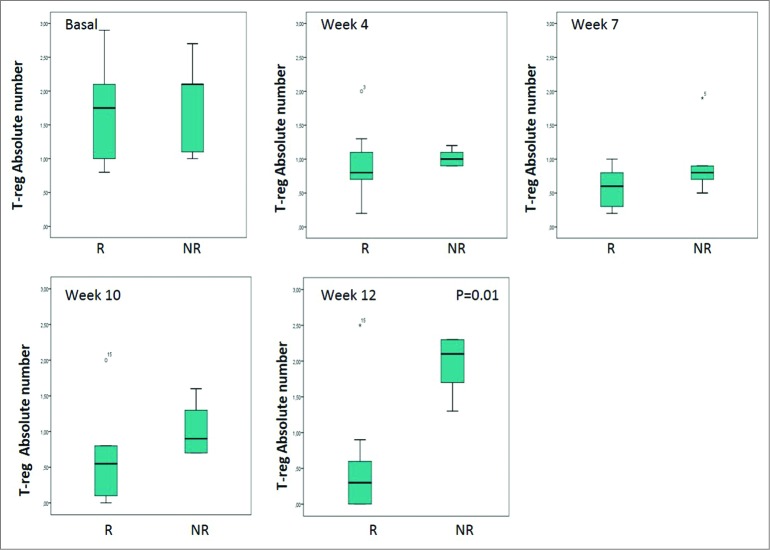
Absolute value of T-reg according to local response. Note: The bottom and top of the box are the first and third quartiles, the band inside is the median, whiskers represents 1.5 interquartile range, points are outliers. R: Responders NR: No Responders.

**Figure 3.**
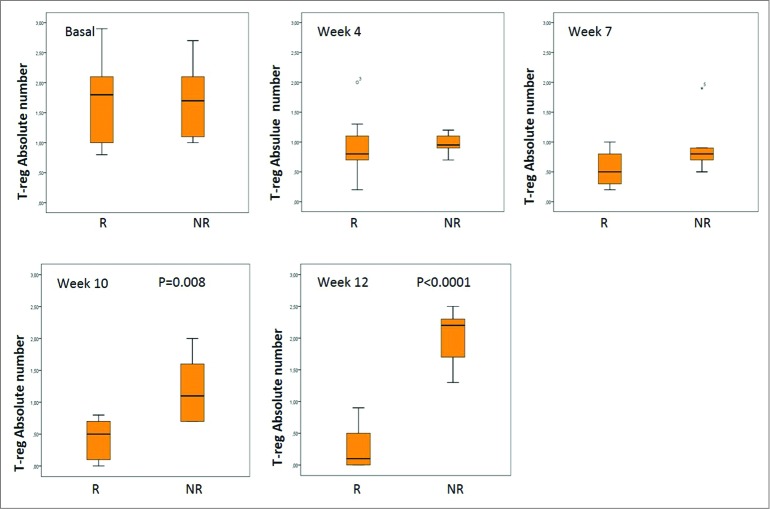
Absolute value of T-reg according to systemic response. Note: The bottom and top of the box are the first and third quartiles, the band inside is the median, whiskers represents 1.5 interquartile range, points are outliers. R: Responders NR: No Responders.

An absolute decrease from baseline in median T-reg cell values was observed in five patients. This decrease ranged from 0.1 to 1.3 and all these patients had a clinical benefit. Median values of –0.7% (–1.6% to 0), –1.1% (–2.1% to –0.2%), –1.1% (–2.2% to –0.7%) and –0.4% (–2.9% to +1.2%) were recorded at Weeks 4, 7, 10, and 12, respectively. A local or systemic response were both associated with significant decreases in T-reg levels compared to baseline. In patients who achieved a local response, the median value of T-reg cells at Week 12 was 0.1 compared with 2.1 in non-responders (**Fig. 2**) (*p* = 0.01). The median T-reg decrease from baseline was also significant at Week 12 for patients who had a local response compared with those without. The difference in the median value of T-reg cells between patients with a systemic response compared with non-responders was significant at Week 10 (*p* = 0.008) and at Week 12 (*p* < 0.0,001) (**Fig. 3**).

No significant difference was observed in the concentrations of CD4^+^ CD25^+^ FoxP3^+^ lymphocytes at Week 12 compared with Week 0 in any of the patients even though five patients had a decrease in circulating T-reg cells. (**Table 3**).

**Table 3.** Concentrations of FoxP3^+^CD4^+^CD25^+^ cells during treatment with ipilimumab and ECT

FoxP3+CD4+CD25+	FoxP3+CD4+CD25+	CD4+tot	CD4+tot
Week 0 (%)	Week 12 (%)	Week 0 (%)	Week 12 (%)
2	3	36.2	26
4	5	54	45
2.1	4.7	27.9	30
3	2.3	34	24
2.1	3.3	35.2	34
2	0.7	50.2	50
0.4	0.6	34	30
3.4	3.7	56	50.2
5	4.5	33.7	30.8
0.4	0.4	53.6	51
6	7.4	25.9	25
5	5.1	31	30
4	4.1	32.8	34.1
0.7	0.6	54	50.3
0.6	0.4	33	30.5

### Safety

Overall this treatment regime was well-tolerated without notable local or systemic toxicity. The most frequent adverse event (AE) was pruritus: grade 1 in nine patients (60%) and grade 2 in three patients (20%). Median time to pruritus onset was 15 d with localization to ECT scar sites observed in seven patients (46%).

## Discussion

This retrospective analysis provides the first reported evaluation of the combination of an approved immuno-oncology agent, ipilimumab, with ECT in patients with advanced melanoma for the treatment of skin lesions. An irDCR of 60% was achieved and of the patients who responded to treatment, 44% patients had SD >3 months. Furthermore, assessment of T-reg cell levels suggests that this parameter could be a potentially predictive biomarker for this therapy combination. These findings require confirmation in additional prospective studies.

It is widely accepted that combination approaches are likely to be required to optimize treatment benefits in advanced melanoma. Two studies with ipilimumab monotherapy both achieved a DCR of 27% (in 155 patients with advanced melanoma ^27^ and 146 patients with advanced melanoma and brain metastases.^28^) Although caution should be exercised when undertaking cross-trial comparisons, a recent study that combined ipilimumab with another immunotherapy, nivolumab, reported an ORR of 40% and a DCR (clinical activity) of 65%.^10^ Ipilimumab has also been combined with other systemic therapies such as dacarbazine (DCR of 33.2%) ^9^ but, in contrast, ipilimumab plus dacarbazine achieved an ORR of just 14.3%.^29^ The efficacy and safety of ipilimumab plus fotemustine was assessed in the NIBIT-M1 Phase 2 trial in patients with metastatic melanoma with or without asymptomatic brain metastases. Overall, patients in this study achieved a DCR of 46.5%: furthermore, those with brain metastases (*n* = 10) achieved a DCR of 50.0%.^30^ The differences in DCR when ipilimumab is combined with different chemotherapies might be explained by different immunogenicity of the combinations. The higher DCR with fotemustine vs. dacarbazine when combined with ipilimumab may be because dacarbazine is less immunogenic. Clearly, not all combinations are going to provide improvements in clinical benefit and each needs to be evaluated to estimate its value.

In addition, combinations of systemic and non-systemic treatment modalities might also be feasible. In a phase Ib study, Puzanov et al. reported that intralesional administration of T-VEC as a priming regimen in combination with ipilimumab resulted in an ORR of 41% with no dose-limiting toxicities in patients with unresected stage IIIB-IV melanoma (*n* = 17).^15^ Similraly, Burnette and colleagues described the potential synergistic relationship between RT and immuno-oncology and proposed that the immunogenicity of RT may be exploited in future treatments.^14,31^ This rationale may also be relevant for ECT.^25^

The clinical importance of ECT treatment and its potential advantages for patients are now recognized. ECT has a high response rate and a favorable safety profile. The limited damage to surrounding tissue coupled with the simplicity of administration and short patient recovery time make it a useful tool for any oncologist.^32^ Studies with non-specific immunotherapies combined with ECT have shown some promise. In a clinical series, Gehl et al. reported that the treatment of melanoma metastatic nodules with ECT and IL-2 resulted in long-lasting remission of distant metastasis in 20% of patients.^33^ The optimal timing of ECT when combined with an immunotherapy is unknown. Based on our retrospective analysis of RT performed after ipilimumab treatment,^14^ it may be better to perform ECT after the first three cycles of ipilimumab (Day 43) in order to exploit the activation of the immune system given by these initial cycles of ipilimumab treatment. However, this needs to be investigated by further studies.

In previous experience, we treated 60 patients with previously untreated cutaneous metastases or in-transit lesions from melanoma with ECT and achieved an ORR of 86.6% (48.4% CR, 38.3% PR).^20^ This compares with a local ORR of 67% achieved in this analysis of patients receiving ipilimumab plus ECT. This lower response can be largely attributed to the different baseline characteristics of the patient cohorts, who were untreated in the previous experience but had recurrent disease after systemic therapy in this analysis. The different biology of the recurrent lesions after treatment could be responsible for the reduced local response. Moreover, any synergism of action between ECT and ipilimumab is likely to be more apparent in improvements in the systemic DCR, rather than changes in local control.

OS was also higher with ipilimumab plus ECT versus our previous experience with ECT alone. With ECT alone, 45 of 74 patients (60.8%) were alive at 6 months, while 30 of 74 (40.5%) patients were alive at 12 months.^20^ This compares with the 6- and 12-month survival reported here of 93.3% and 86.2%, respectively.

There are reports, albeit limited, of an abscopal effect being observed in patients treated with ipilimumab and RT. The abscopal response has been reported with treatment of melanoma, Merkel cell carcinoma, hematologic malignancies and solid tumors. It is an unusual response to RT in which radiation of a tumor causes regression of untreated distant skin lesions, with the mechanism not well-defined. However, one study performed with monoclonal antibodies to analyze infiltrating lymphocytes suggested that the abscopal effect might be due to cellular immunity activation.^12-14^

Several reports support the hypothesis that ECT can induce anticancer immunity. In melanoma, which is considered among the most immunogenic of tumor types, ECT combined with IL-2 induced tumor cell death.^33^ To understand if the local response triggered by ECT may be used to elicit a systemic response, Gerlini and colleagues ^34^ focused on the timing of the sensitization of antigen-presenting cells to tumor antigens at the site of treatment. Repeated histological tests at melanoma ECT-treated nodules suggested that 7 d post-treatment there were no Langerhans cells within the nodule, while at Day 14 activated cells were present displaying dendritic morphology. The presence of plasmocytoid dendric cells (pDCs) expressing TLR7 and TLR9 receptors in the inflammatory infiltrate of melanoma metastasis is crucial for treatment with two potent immune stimulators after ECT, such as imiquinod, a TLR7 agonist, and GpG-ODN, a TLR9 agonist. The blockade of the immune system by regulatory checkpoints such as ipilimumab might represent an alternative strategy to enhance immunity.^34^ Combined RT and immunotherapy with ipilimumab showed a synergistic effect with regression of non-irradiated distant lesions (abscopal effect).^13,14^ The blockade of immune checkpoints might also be able to improve and extend the beneficial effects of ECT.

As ECT-based technology evolves, it will be interesting to see the impact this has on future treatment regimens in oncology. This technology may eventually provide multiple clinical applications. Continuous technological development and clinical investigation of electroporation-based treatments will only serve to further increase its clinical relevance.

Clinical trials have demonstrated that it is difficult to achieve a correlation between immunological data and clinical outcomes. There is a need to identify those patients that will respond to ipilimumab therapy and to establish a method by which they can be monitored during treatment. The development and validation of such biomarkers would provide clinical advantages. A relationship between absolute lymphocyte count and clinical benefit with ipilimumab has been reported, as well as increased expression of inducible costimulator by CD4^+^ T cells in peripheral blood and tumor tissue resulting in an increase in the ratio of effector to regulatory T cells after ipilimumab treatment.^35,36^

In our cohort of patients treated with ipilimumab in the EAP, we explored the possible prognostic or predictive value of some potential biomarkers.^26^ Circulating T-reg cells were part of such analysis. We analyzed this lymphocytes population to establish whether their circulating levels could be used as predictive biomarker for response. This analysis was performed on 95 patients treated with ipilimumab, and showed that a reduction or no change in LDH, CRP and FoxP3/T-reg cells between baseline and Week 12 was associated with improved survival of patients treated with ipilimumab, suggesting that the evaluation of circulating FoxP3/Treg cells could represent a possible prognostic marker.^26^ In the current analysis, T-reg levels decreased in all responders and were significantly lower than in non-responders at Week 12. Further studies are needed to validate this relationship.

Ipilimumab is associated with immune-related AEs, commonly affecting the skin and gastrointestinal tract.^8,37^ Among our patients, 80% experienced pruritus (grades 1–2). Other typical side effects of ipilimumab, such as diarrhea, were not observed, probably because of the small number of patients considered in this analysis. The safety profile of ipilimumab registered in the Italian EAP was previously reported and was consistent with that observed in clinical trials.^38^ ECT is not associated with serious AEs,^39^ and is therefore not likely to increase the risk of significant toxicity in this treatment regimen.

It should be noted that our analysis has some limitations: its retrospective nature, the small number of patients considered, the use of ECT only on cutaneous and subcutaneous lesions with the absence of data about internal lesions. We cannot confirm whether the ipilimumab and ECT combination had an additive effect, or whether a similar disease course could have been achieved with ipilimumab treatment alone. However, this preliminary analysis suggests that a combination approach of ipilimumab followed by ECT is feasible with a manageable safety profile and may increase the therapeutic response in skin lesions. Based on this retrospective analysis, this combination was beneficial to patients with advanced melanoma and this novel treatment regimen warrants further investigation in prospective clinical trials.

### Patients and methods

The ipilimumab EAP included patients aged ≥16 years who had histologically confirmed, measurable (using modified World Health Organization [WHO] criteria) stage III (unresectable) or stage IV melanoma who had progressed during or after at least one prior therapeutic regimen containing one or more of interleukin-(IL)-2, dacarbazine, fotemustine, or temozolomide. Patients were required to have a life expectancy of ≥16 weeks and an Eastern Cooperative Oncology Group performance status of 0–1.^40^

For patients who met all the EAP inclusion criteria^40-44^ and who had no alternative treatment option available, physicians were able to request ipilimumab. The EAP was approved by a local ethics committee/institutional review board (IRB).^38^ All patients enrolled in the EAP provided signed informed consent before treatment with ipilimumab. In addition, patients provided written consent before receiving ECT. Ethics committee/IRB approval for the occasional use of ECT was not considered necessary because this was not a clinical trial. All patients treated at our research institute also sign a standard informed consent form at their first visit to allow blood sampling.

Among 120 patients that met the EAP inclusion criteria^40-44^ and were treated with ipilimumab, 15 patients were considered suitable for palliative loco-regional control with ECT (due to pain and/or bleeding of lesions). Patients received ECT if they had cutaneous or subcutaneous lesions that were accessible for the application of electric pulses using single-use, sterile Cliniporator™ electrodes. For patients presenting with more than seven lesions, the lesions with the largest diameters (3–30 mm) were considered ‘target’ lesions. Patients with any serious coagulation abnormality, symptomatic congestive heart failure, pulmonary embolism within the 6 months prior to study drug administration), severe chronic bronchopneumonitis, known allergy to bleomycin, cumulative lifetime dose of bleomycin exceeding 250 mg/m^2^, peripheral neuropathy greater than grade 2, or epilepsy were not considered suitable for ECT. Pregnant or breastfeeding women were also not considered for ECT.

Patients were treated with ipilimumab 3 mg/kg as an intravenous infusion every 3 weeks for four cycles and followed for a further 12 weeks (a total 24-week study period). No dose variation was performed. Criteria for treatment interruption and dose reductions were those commonly used for ipilimumab. ECT was administered after the first ipilimumab infusion. ECT consisted of delivering high-voltage pulses from a Cliniporator™ (IGEA S.p.A., Italy) to the target lesions 8 min after intravenous administration of bleomycin (15 mg/m^2^). ECT was applied according to validated European Standard Operating Procedure of ECT (ESOPE).^16,17^

#### Assessments

The main objective of this retrospective analysis was to evaluate the benefit of ECT after ipilimumab treatment for patients in terms of irDCR. Evaluation of a potential predictive biomarker (T-reg cell levels) for response to this treatment combination was also performed.^26^

The proportion of patients with a local CR, PR, or SD according to WHO criteria was measured at the end of the study period. The first local response assessment was performed at Week 2, then at Weeks 4 and 8. Response was evaluated according to WHO criteria. Systemic response assessments were performed at Week 12 and every 12 weeks. If PD was noted, a further evaluation was performed after 4 weeks. Systemic response was evaluated according to irRC.^27^

Blood samples were collected before ipilimumab, after ipilimumab and before ECT, plus 1, 15 and 30 d following ECT, at each cycle of ipilimumab and every 12 weeks (tumor assessment time point). Isolated peripheral blood mononuclear cells (PBMC) were labeled with anti-CD4-Pe-Cy-5, CD25-Pe and anti-FoxP3-AlexaFlour488 to identify circulating T-reg cells. Concentrations of CD4^+^ and CD25^+^ cells and T-reg levels were measured using a flow cytometer assay for PBMC in CD4^+^ cells. Cells, stained with antibodies against FoxP3/CD25, were identified according to the expression of CD4^+^, CD25hi and FoxP3^+^ by fluorescence-activated cell sorting (FACS) using the FACS ARIA II flow cytometer system and FACS Diva™ software (BD Biosciences; Mountain View, CA, USA).

Possible biomarkers were evaluated using blood samples. This was part of a more extensive analysis previously reported^26^ with the main aim being to assess whether the addition of ECT to ipilimumab had any notable effect on biomarker evaluation.

#### Safety

AEs were graded using the National Center Institute–Common Terminology Criteria (version 3.0). Frequency and severity of all AEs were recorded for all patients receiving at least one dose of ipilimumab.

#### Statistical analyses

Data were summarized according to descriptive methods: absolute frequencies and percentages were reported for categorical variables, median and range for continuous items. Differences in distributions according to response were tested using a non-parametric approach (Mann–Whitney test).
